# Patterns of transcriptional parallelism and variation in the developing olfactory system of *Drosophila* species

**DOI:** 10.1038/s41598-017-08563-0

**Published:** 2017-08-18

**Authors:** Jia Wern Pan, Qingyun Li, Scott Barish, Sumie Okuwa, Songhui Zhao, Charles Soeder, Matthew Kanke, Corbin D. Jones, Pelin Cayirlioglu Volkan

**Affiliations:** 1 Department of Biology, Duke University, Durham, North Carolina USA; 2 Department of Biology, Stanford University, Stanford, California USA; 3 Pratt School of Engineering, Duke University, Durham, North Carolina USA; 4 Department of Biology and Integrative Program for Biological & Genome Sciences, University of North Carolina at Chapel Hill, Chapel Hill, North Carolina USA; 5 Department of Genetics, University of North Carolina at Chapel Hill, Chapel Hill, North Carolina USA

## Abstract

Organisms have evolved strikingly parallel phenotypes in response to similar selection pressures suggesting that there may be shared constraints limiting the possible evolutionary trajectories. For example, the behavioral adaptation of specialist *Drosophila* species to specific host plants can exhibit parallel changes in their adult olfactory neuroanatomy. We investigated the genetic basis of these parallel changes by comparing gene expression during the development of the olfactory system of two specialist *Drosophila* species to that of four other generalist species. Our results suggest that the parallelism observed in the adult olfactory neuroanatomy of ecological specialists extends more broadly to their developmental antennal expression profiles, and to the transcription factor combinations specifying olfactory receptor neuron (ORN) fates. Additionally, comparing general patterns of variation for the antennal transcriptional profiles in the adult and developing olfactory system of the six species suggest the possibility that specific, non-random components of the developmental programs underlying the *Drosophila* olfactory system harbor a disproportionate amount of interspecies variation. Further examination of these developmental components may be able to inform a deeper understanding of how traits evolve.

## Introduction

The *Drosophila* genus of fruit flies includes 1500 species inhabiting a variety of niches^1–3^ and exhibits a diverse array of odor-guided behaviors with both conserved^4–6^ and adaptable^7–9^ components. The *Drosophila* olfactory system is a fast-evolving system^7–9^ consisting of a highly diverse set of olfactory receptor neuron (ORN) classes that interact with and decipher a complex chemical environment^10^. The primary olfactory sensory appendage in adult *Drosophila* fruit flies is the third segment of the antenna, which is covered with multiporous sensory hairs called “sensilla”. There are three major morphological types of sensilla: club-shaped basiconica (ab: antennal basiconic), spine-shaped trichoidea (at), and cone-shaped coeloconica (ac), each with a stereotypical distribution on the surface of the antennae. Each morphological sensilla type can be further broken down into a few different sensilla subtypes, which are defined by the unique combination of the olfactory receptor neuron (ORN) classes that they house. Each sensillum houses a small number (1–4 in *D*. *melanogaster*) of ORNs, which typically expresses a single receptor gene from a large genomic repertoire and connects to a stereotypical glomerulus in the antennal lobe of the brain^11–13^.

The olfactory system can be critical to the evolution of new food specializations. Close relatives of the popular model species *D*. *melanogaster*, *D*. *erecta* and *D*. *sechellia* have independently evolved to become specialists on different host plants; both have a narrow host range and show a strong preference for odorants from their respective host plants, including some odorants that are normally repulsive to other closely-related generalist species^8,9^. These two species are a striking example of parallel evolution of the olfactory system. During the process of specialization on new host plants — and accompanying chemosensory behavioral shifts — the olfactory neuroanatomy of both species have evolved in similar ways^8,9,14^. In both *D*. *erecta* and *D*. *sechellia*, increased sensitivity to their respective host volatiles appears to be modulated by the ORNs expressing the exact same odorant receptor, *Or22a*. In addition, in both *D*. *erecta* and *melanogaster-sechellia* hybrids, there is an increase in the number of the antennal ab3 sensilla subtype that houses the *Or22a* ORNs, along with an increase in the volume of the *Or22a* glomerulus. However, other than the characterizations of these structural components that have elucidated these similarities, not much is known about the genetic mechanisms giving rise to these parallelisms in the developing and adult peripheral olfactory system.

The developmental programs regulating the diversity of neuronal fates in the peripheral olfactory system are complex and are only partially identified. In an attempt to disentangle the multifactorial basis of the neuroanatomical convergence in the adult olfactory system, one needs to consider the genes, especially transcription factors (TFs), that direct the developmental process giving rise to antennal structures. In fruit flies, the adult antennae arise from the eye-antennal discs in the developing larvae. Recent studies on *D*. *melanogaster* have shown that the development of the *Drosophila* peripheral olfactory system can be divided into three stages. First, in the developing antennal disc, combinations of transcription factors belonging to a gene regulatory network initiated by morphogen gradients demarcate the antennal disc into several concentric rings, each with a unique differentiation potential^15^. Next, multipotent sensory organ precursors are selected within the rings by the expression of proneural TFs such as Amos and Atonal^16,17^. Finally, each precursor then undergoes Notch-mediated asymmetric cell divisions, facilitated by yet another set of terminal selector TFs, to generate and diversify both the ORN and non-neuronal components of each sensilla subtype^18–20^.

Based on the knowledge of the structure^11,13,21,22^ and development^15–20,23^ of the olfactory system in *D*. *melanogaster*, the parallel evolution of *D*. *erecta* and *D*. *sechellia* suggests that the adaptation of olfactory circuit responses to a specific host odor in specialists follows a common development trajectory, at least in some cases. Given that the relationships between specific olfactory receptors and specific developmental transcription factors are well-characterized in *D*. *melanogaster*, we asked whether similar parallelisms between *D*. *sechellia* and *D*. *erecta* result from parallel changes in their respective development transcriptional programs.

In this study, we investigate the extent of parallelism in the adult and developing olfactory system of specialist *Drosophila* species, as well as attempt to identify potential developmental regulators of this parallelism using transcriptomics and gene specific expression analysis. Targeting the adult antennae and the three key developmental stages described above, we compare RNAseq data from the two specialist species and four related generalist Drosophilids. This taxonomic sampling enables us to contrast what is occurring in each specialist lineage in a phylogenetically aware way.

Here we report that transcriptional programs operating during development in the two specialist species—predominantly associated with a subset of transcription factors—are strikingly parallel, unpredicted from their phylogenetic relationship. This is in contrast to more constrained parallelism in adult olfactory receptor expression in the two species. Comparing the transcriptional profiles with general patterns of variation in the adult and developing olfactory system of the six species showed that a subset of olfactory receptor genes expressed particularly in basiconic sensilla olfactory receptor neurons^11,13,21,22^, along with a disparate subset of the transcription factors governing olfactory system development^15–17,20,23^, are disproportionally variable across all six *Drosophila* species relative to other olfactory receptor neuronal lineages, implying the presence of non-random variation within both the adult and developing *Drosophila* olfactory system. We hypothesize that the developmental parallelisms in specialist species and the non-random patterns of variation in the adult and developing antennae may arise from a limited number of “flexible” molecular components during development.

## Results

**Figure 1:**
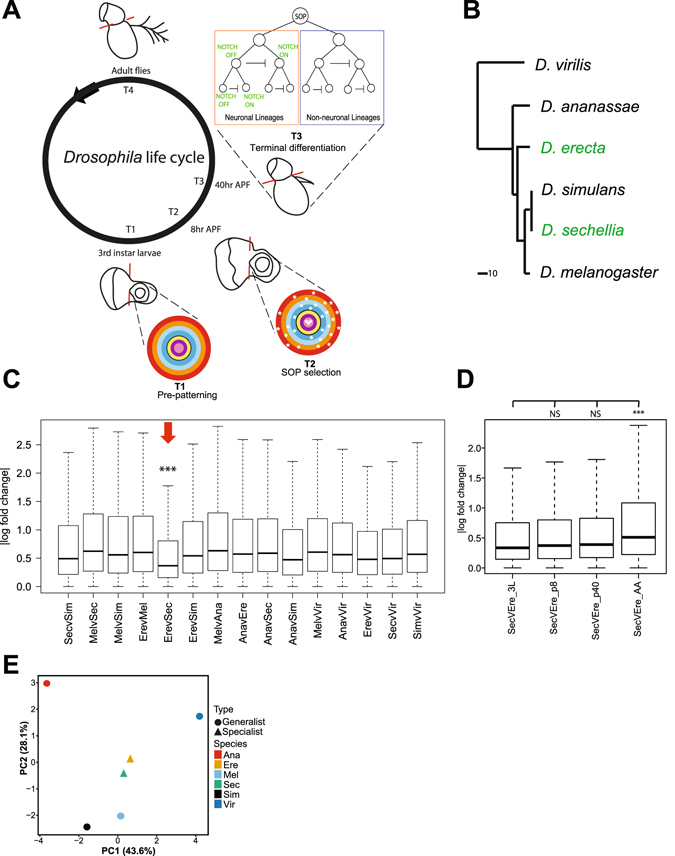
Broad parallelism in *D*. *erecta* and *D*. *sechellia* antennal transcriptomes during development. **(A)** Developmental time points during which samples were collected. The dotted red lines indicate where the antennal disc and the third antennal segment were isolated from surrounding tissue. Also shown are schematics of the developmental stages corresponding to each time point. **(B)** Phylogenetic tree of the six *Drosophila* species represented in our study^2,3^, with the positions of the specialist species *D*. *sechellia* and *D*. *erecta* highlighted in green; the scale bar represents time since divergence in millions of years (mya). **(C)** Pairwise interspecies comparisons of absolute log fold change (ALFC) values for all genes across all developmental time points, normalized for phylogenetic distance (see Methods). Asterisks indicate that *D*. *sechellia* versus *D*. *erecta* ALFC is significantly different compared to all other columns (ANOVA + Tukey’s HSD, n > 29,000, p < 0.001). **(D)** Comparisons of *D*. *sechellia* versus *D*. *erecta* ALFC for all genes across different developmental stages (ANOVA + Tukey’s HSD, n > 7000, NS = not significant, ***p < 0.001). **(E)** Principal component analysis (PCA) of DESeq-normalized transcript counts for transcription factors with known involvement in the development of the olfactory system (Table S1) during the 3^rd^ instar larval (3L) stage (n = 21).

The specialists *D*. *erecta* and *D*. *sechellia* are members of the *melanogaster* group, along with the model genetic organism *D*. *melanogaster*, which is the fly whose olfactory system is best understood (Fig. 1A). (For simplicity, all timing and description below is put in terms of *D*. *melanogaster* development, anatomy, and annotation.) In order to analyze species-specific transcriptional variation, we compared the transcription profiles of the adult and developing antennae from the two specialist species *D*. *sechellia* and *D*. *erecta*, along with *D*. *melanogaster* and three other generalist species, *D*. *ananassae*, *D*. *simulans*, and *D*. *virilis*. *D*. *simulans* and *D*. *ananassae* represent species that are closely related the specialists but do not evince specialization. We included *D*. *virilis* as a distant outgroup (Fig. 1B).

As the development of the *Drosophila* peripheral olfactory system has three distinct phases representing fundamental transitions in antennal development, we sampled RNA from each of these points and adults. Specifically, we profiled transcripts from antennal discs at third instar larvae (3L) (T1) and 8 hours after puparium formation (APF) (T2), as well as from antenna at 40 hours APF (T3) and in adults (T4). These time points correspond to the developmental stages of prepatterning, precursor selection, onset of olfactory receptor (OR) expression and wiring of olfactory receptor neurons (ORNs), and the final differentiated adult antennae, respectively (Fig. 1A)^15^. All samples were stage matched across species. Each sample consisted of a pool of ~70 larvae/pupae or ~300 adult flies according to developmental time, and we had two biological replicates for each sample.

### Developmental parallelisms in specialist *Drosophila* species

From the RNAseq data, we first analyzed the extent of parallelisms in the antennal transcription profiles for the two specialist *Drosophila* species. A challenge for comparing transcription across species and genes are differences in baseline expression and magnitude—a modest change in one gene may be equivalent to a massive change in another. Acknowledging this concern, we focused on the magnitude of change relative to baseline and following Enard *et al*.^24^, calculated and compared the absolute log fold change (ALFC) in gene expression for each gene across all pairwise comparisons. ALFC captures the variability of a gene across evolutionary time scales: a high ALFC value represents a gene that has high transcriptional variability across the species examined, while a low ALFC value represents a gene with relatively conserved levels of transcription across those species.

Contrary to “neutral” phylogenetic expectations, we found that pairwise comparisons of *D*. *sechellia* and *D. erecta* had the lowest pairwise ALFC values across all genes and developmental time points – that is, gene expression in the developing and adult antennae is more similar between these two species compared to other closely related species (Fig. 1C). Importantly, these results indicated that the parallelisms between *D. sechellia* and *D. erecta* are highly polygenic and not restricted to one gene or gene network. Our results were consistent with known structural similarities in ORN distributions associated with host plant specialization^8,9^, but also suggested a potential broader evolutionary parallelism during transcription.

Developmental stage-specific analysis of pairwise ALFC showed that the similarity between *D. sechellia* and *D. erecta* transcription profiles appears to be greater during the developmental stages (3L through p40) compared to adult antennae (Fig. 1D), suggesting that these parallelisms are more apparent during development than in the adult antennae (see also Supp. Fig. 1 for the broader interspecies context of these pairwise comparisons). For example, we found TFs with known roles in development of specific ORNs among the top 50 most convergent genes from 250 known transcription factors in *D. sechellia* and *D. erecta* (Fig. S2). Principal component analysis (PCA) of all TFs known to be responsible for patterning ORN precursors^15–17,20^ during the 3^rd^ instar larval stage also indicated that the transcription profiles for these TFs are more similar between the two specialists species than with any other species (Fig. 1E). This similarity in the transcriptional profiles of specialists suggests that the extent of parallelism is wider than previously reported and includes known ORN developmental programs, which in turn suggests parallel shifts in the molecular networks governing the configuration of the adult olfactory system.

### Validation of interspecies transcriptional variability in adult and developing antennae

**Figure 2:**
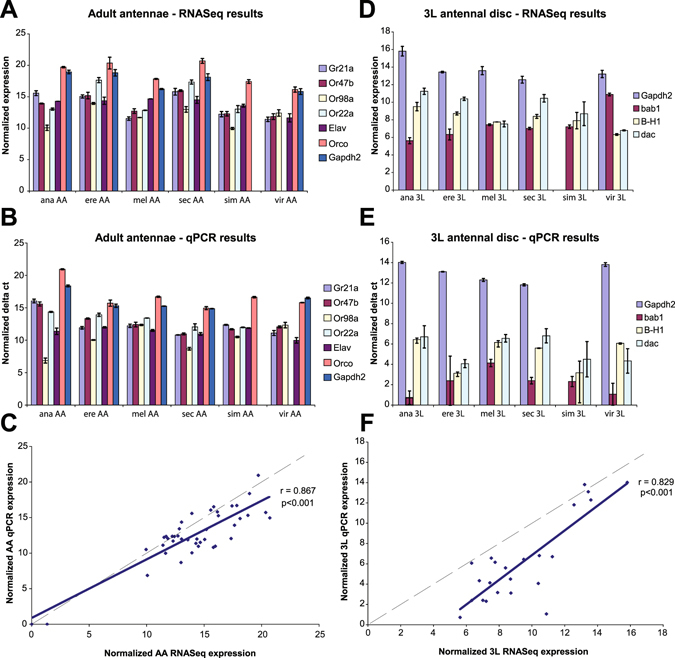
qPCR validation of RNASeq results. **(A)** RNASeq expression values for select genes from the adult antennae, log normalized against *Act5C*, and **(B)** corresponding qPCR delta Ct values for the same genes from the adult antennae, also normalized against *Act5C*. **(C)** Plot of normalized RNASeq results versus normalized qPCR results (n = 42). The dotted line indicates where x = y, and the solid line shows a fitted line, with r as Pearson’s correlation coefficient. **(D)** RNASeq expression values for select genes from the 3^rd^ instar larval antennal disc, log normalized against *Act5C*, and **(E)** corresponding qPCR delta Ct values for the same genes from the 3L antennal disc, also normalized against Act5C. **(F)** Plot of normalized RNASeq results versus normalized qPCR results (n = 24). The dotted line indicates where x = y, and the solid line shows a fitted line, with r as Pearson’s correlation coefficient. Error bars indicate the 99% confidence interval.

To confirm the results from our transcription profile comparisons, we validated our RNASeq results for a subset of genes using quantitative RT-PCR (qPCR) as well as a combination of fluorescent RNA *in situ* hybridization, genetic markers in *melanogaster* hybrid flies, and antibody stainings. Transcription profiles using qPCR are difficult to compare between species, as this would require the assumption of equal levels of transcription for control housekeeping genes. However, qPCR profiles do allow for good relative comparisons of the transcription levels for different genes within a single species, which we can compare with our RNASeq results for those genes that have normalized in an equivalent manner. As such, we selected a random subset of olfactory receptor (OR) genes from the adult antenna and a smaller random subset of pre-patterning TF genes from the 3L antennal disc, along with a few housekeeping genes from both time points for qPCR validation. Our results from qPCR analysis of the adult antenna were tightly correlated with our RNASeq results across all examined genes (Fig. 2A–C), providing support for the reliability and accuracy of our RNASeq data. Likewise, our qPCR results from the 3L antennal disc also had a high, statistically significant correlation with our RNASeq results (Fig. 2D–F), although in this case there appeared to be slightly greater discrepancy. Overall, we believe that our qPCR data supported the accuracy and reliability of our RNASeq data and thus provided indirect support for the conclusions drawn from that dataset.

**Figure 3:**
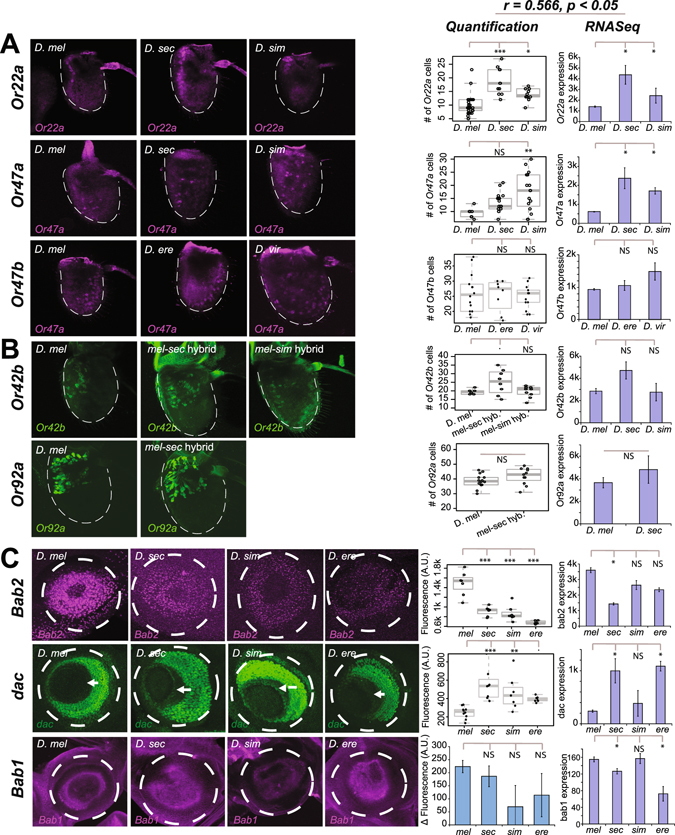
Validation of RNASeq results with *in vivo* experiments. **(A)** Fluorescent RNA *in situ* hybridization for *Or22a*, *Or47a* and *Or47b* genes in the antenna across multiple species. **(B)** Reporter analysis for *D*. *melanogaster* hybrid F1 crosses carrying promoter-fusion GFP constructs for *Or42b* and *Or92a* in the antenna. *D*. *mel*. – *D*. *sim*. hybrid crosses for *Or92a* did not produce any progeny. **(C)** Antibody stainings for the Bab2 and Dac proteins, and RNA FISH for *bab1*, in the 3^rd^ instar larval antennal disc across multiple species. White arrows in *dac* images indicate expansion of *dac* towards the central fold in some species. Quantification for *dac* is for an area within the zone of expansion. Quantification for *bab1* is for the difference in fluorescence of the antennal disc between antisense and sense probes. Statistical comparisons for *in vivo* comparisons shown are ANOVA + Tukey’s HSD, which controls for family-wise false discovery rate (p < 0.1, *p < 0.05, **p < 0.01, ***p < 0.001, NS = not significant), except for *Or92a* which shows the uncorrected results for a Student’s T-test. RNASeq figures show DESeq-normalized transcript counts, with error bars indicating S.E.M., and significant comparisons shown are for DESeq. 2-calculated adjusted p-value using stringent criteria (*p < 1E-8). FDR was controlled for using Benjamini-Hochberg method. Also shown is the correlation between predicted RNASeq pairwise log fold change in gene expression and the corresponding log fold change in the quantification of *in vivo* comparisons (see Fig. S3).

The changes in gene expression observed in RNAseq data for specific genes can be an outcome of changes in the levels of expression across cells and/or changes in the number of cells expressing that gene. To look for *in vivo* changes in gene expression levels and patterns for a number of genes across some of the species included in our study, we performed *in situ* hybridizations, reporter analysis in hybrid crosses, and immunohistochemistry experiments when applicable. Using fluorescent RNA *in situ* hybridization, we were able to count the number of cells in the antennae that expressed the odorant receptors Or22a, Or47a, or Or47b and found species-specific differences that were mostly consistent with the differences that we saw in our RNASeq data (Fig. 3A). Specifically, we found that both *D. sechellia* and *D. simulans* showed significant increases in the number of Or22a ORNs compared to *D. melanogaster*, as previously reported^7,25^, and there were significantly more Or47a ORNs in *D. simulans* than *D. melanogaster* (Fig. 3A). We also detected a small increase in Or47a ORNs in *D. sechellia*, but this was not statistically significant (Fig. 3A). Additionally, the number of Or47b ORNs was not significantly different across species, similar to our RNASeq data (Fig. 3A). Similarly, counts for Or42b and Or92a in *sechellia-melanogaster* hybrids carrying fluorescent genetic markers for those genes also showed patterns that were consistent with species differences in the RNASeq data (Fig. 3B).

We also used antibody stainings on 3L antennal discs to analyze the expression pattern of Bab2 and Dac proteins, which are important regulators of olfactory system development and show transcriptional variation across species. In *D. melanogaster*, Bab2 is normally expressed in a gradient within the antennal disc, and Bab2 expression levels appear to be decreased in *D. simulans*, *D*. *sechellia* and *D*. *erecta* (Fig. 3C). The species-specific differences in protein levels of Bab2 were confirmed by quantification of the changes in antibody fluorescent intensity across the antennal disc, and were consistent with differences in transcription between species detected in RNASeq. Whole-mount RNA FISH for *bab1* also yielded a similar pattern (Fig. 3C). On the other hand, in *D*. *melanogaster*, Dac is expressed in the outer discs, with no expression near the central fold (Fig. 3C). However, in *D*. *sechellia*, and more prominently in *D*. *simulans* and *D*. *erecta*, Dac expression appears to expand to a new ring of single cell thickness adjacent to the central fold (arrows in Fig. 3C). This pattern is consistent with our RNASeq results for *D*. *sechellia* and *D*. *erecta* but not for *D*. *simulans*, although the inconsistency with RNASeq results for *D*. *simulans* may be due to a decrease in overall *dac* transcription in *D*. *simulans* separate from the expansion.

To directly compare our RNASeq results to *in vivo* differences, we asked if our RNASeq results were correlated with changes in RNA FISH, hybrid crosses and imunofluorescence. To do so, we compared the interspecies pairwise log fold change in gene expression predicted by RNASeq with the corresponding pairwise log fold change in the quantification of the *in vivo* results and found that RNASeq differences were significantly correlated with the *in vivo* differences we detected (Pearson’s r = 0.566, df = 18, p < 0.05) (Fig. S3). Thus, even though there are some discrepancies among specific individual comparisons, taken as a whole these results show that the RNASeq data in general capture the differences in ORN composition we observe among species. The discrepancies, such as *Or47a*, may be due to a disconnect between the amount versus the pattern of transcription or post-transcriptional processing of the transcript. While understanding the basis of these discrepancies will require further work, our data suggest RNAseq is a reasonable proxy for ORN expression patterns.

### Transcriptional variability and parallelism in the adult olfactory system

Next, we used our RNASeq dataset to test a hypothesis regarding the evolution of the *Drosophila* olfactory system: that components with greater flexibility are more likely to be utilized during adaptive events. To test this hypothesis, we focused specifically on OR genes in the adult stage and TFs with known roles in ORN development.

**Figure 4:**
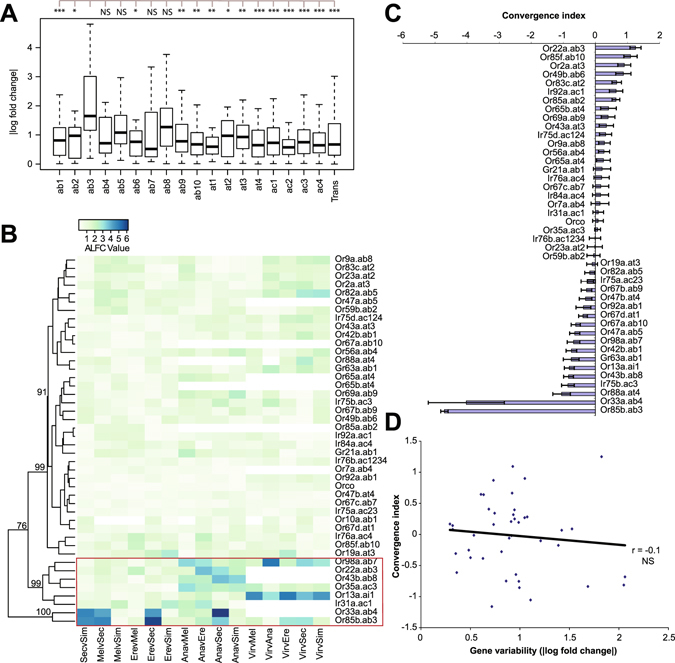
Transcriptional variability of olfactory receptors across six *Drosophila* species. **(A)** Boxplot and (**B**) heatmap plotting the absolute log fold change (ALFC) in the expression of olfactory receptors (ORs) across all pairwise interspecies comparisons. The boxplot shows combined values for each sensilla subtype, and includes values for the entire antennal transcriptome (Trans) for comparison. Statistical comparisons show results for ANOVA + Tukey’s HSD (*p < 0.05, **p < 0.01, ***p < 0.001, NS = not significant). The post-hoc test compares ab3 AFLC to the other classes of ORs. A non-parametric Kruskal-Wallis H test for the boxplot also had p-value < 0.001 (df = 5). In the heatmap, invalid comparisons without comparable orthologs (NAs) are white/blank, highly variable genes are highlighted in red, and numbers indicate bootstrap support for the clustering analysis. **(C)** Convergence index values (see Methods) showing levels of convergence between *D*. *sec*. and *D*. *ere*. for olfactory receptor genes in the antenna. Positive values indicate convergence while negative values indicate divergence. Error bars indicate S.E.M. **(D)** Plot of convergence index values versus absolute log fold change (ALFC) values for antennal olfactory receptor genes. ALFC values shown are the mean ALFC value across all interspecies pairwise comparisons. The solid line shows a fitted line, with *r* as Pearson’s correlation coefficient.

Focusing first on the adult stage, we first looked for differences in flexibility among the OR genes. In order to quantify transcriptional variability in OR genes, we analyzed the absolute log fold change (ALFC) values across all pairwise species comparisons for OR transcripts in the adult antennae. We first plotted these ALFC values as a boxplot showing the mean and quartile values for all OR genes grouped according to sensilla subtype (Fig. 4A). Contrary to our expectations, we found that interspecies variability in OR genes was not randomly distributed. OR genes expressed in ORNs housed mostly in the basiconic sensilla subtypes (particularly ab3) appeared to have significantly more transcriptional variability across species than others, while most other subtypes were not significantly more variable than the whole antennal transcriptome (Fig. 4A).

To examine these patterns of variation in more detail, we plotted each pairwise interspecies ALFC value for each individual OR gene as a heatmap, with a range from lighter green to darker blue indicating lower and higher variation, respectively. Clustering analysis of this heatmap revealed a small subset of adult OR genes that appeared to be substantially more variable than other OR genes, which in general showed relatively stable levels of transcription across species (Fig. 4B). This subset of OR genes included OR genes normally expressed in ORNs housed in basiconic sensilla, specifically *Or22a* (ab3), *Or85b* (ab3), *Or33a* (ab4), *Or98a* (ab7), and *Or43b* (ab8). *Or13a* and *Ir31a*, expressed by ORNs in the intermediate and coeloconic sensilla, respectively, also show transcriptional variation. Importantly, the variability across species is not exclusively linked with specialist vs. non-specialist comparisons, suggesting that this subset of ORs is distinctly variable relative to the strong homogeneity seen among all other ORs. As a whole, these results suggest that transcriptional variability in OR genes is mostly limited, but not restricted, to specific ORN lineages from the basiconic class of sensilla.

Behavioral adaptations seen in *D*. *sechellia* and *D*. *erecta* have previously been tied to parallel transcriptional or structural reconfigurations of the adult olfactory system in these two species, particularly defined by a parallel increase in *Or22a* ORN numbers housed in basiconic sensilla^8,9^. Thus, it is possible that highly variable ORN lineages are more likely to contribute to adaptive chemosensory/behavioral evolution. As such, we expected some of the OR genes that show high amounts of variation among the six species to also be highly convergent between *D*. *erecta* and *D*. *sechellia*. In order to test this hypothesis and identify other parallel changes, we ranked the adult OR genes for those that appear most similar between the specialists *D*. *erecta* and *D*. *sechellia* relative to (i.e. normalized by) sister species with equivalent divergent time estimates. We summarized these into a single statistic as the “convergence index”. High, positive values for the convergence index represent genes that are highly converged in *D*. *erecta* and *D*. *sechellia*, while low, negative values represent genes that are highly divergent in these two species relative to other species with equivalent divergent time estimates. As expected, among the adult antennal OR genes, the gene with the highest convergence index value was the *Or22a* (ab3) gene (Fig. 4C), which has been implicated in detecting odors from the host plants of the two specialists, *D*. *sechellia* and *D*. *erecta* ^7–9^. *Or22a* normally exhibits high interspecies variation in transcript levels (Fig. 4B), but shows little variation between *D*. *erecta* and *D*. *sechellia* suggesting convergent transcript levels in specialists (Fig. 4C). Thus, our data confirms previous morphological findings at the transcriptional level suggesting similar configurations of the same flexible component in the two specialist species. However, not all genes with high transcriptional variation across species show high convergence index values. An example of this is *Or85b*, which shares the ab3 sensillum with Or22a ORNs, and also shows high transcriptional variability across 6 species. *Or85b* and *Or22a* levels in *D*. *erecta* show a concomitant increase compared to *D*. *melanogaster* (Fig. S4). However, *D*. *sechellia*, which has augmented *Or22a* levels, shows a decrease in *Or85b* expression (Fig. S4). These differences between *Or22a* and *Or85b* might reflect differences in species-specific developmental programs driving sensilla identity, as well as the OR choice in each ORN housed in that sensilla. Additionally, a plot of gene variability versus convergent index values showed no significant relationship between gene variability and convergence for adult OR genes, suggesting that even though some OR genes with convergent profiles have high evolutionary variability, variable OR genes are (with a few exceptions) not more convergent in specialist species. This low-level convergence in OR expression is consistent with our previous results indicating fewer parallelisms in the adult stage compared to earlier developmental stages (also see below) (Fig. 1D). Nevertheless, ecological and species-specific constraints may still cause specific OR genes from more variable OR transcriptional outputs to be selected for and utilized during specific adaptive events.

### Transcriptional variability and parallelism in antennal developmental programs

**Figure 5:**
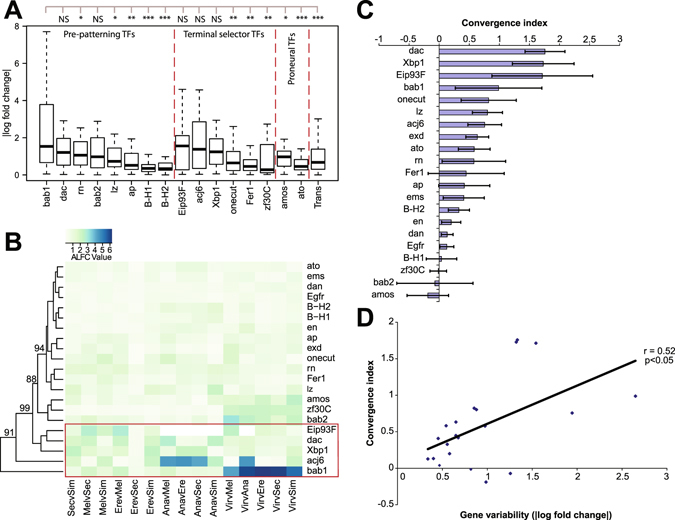
Transcriptional variability of antennal developmental transcription factors across six *Drosophila* species. **(A)** Boxplot and (**B**) heatmap plotting the absolute log fold change (ALFC) in the expression of transcription factors known to specify the adult olfactory system^15–17,20^ across all pairwise interspecies comparisons and across all three developmental time points (T1–T3). The boxplot shows combined values for each transcription factor across development, and includes values for the entire antennal transcriptome (Trans) for comparison. Statistical comparisons show results for ANOVA + Tukey’s HSD (*p < 0.05, **p < 0.01, ***p < 0.001, NS = not significant). The post-hoc test compares bab1 AFLC to the other TF. A non-parametric Kruskal-Wallis H test for the boxplot also had p-value < 0.001 (df = 5). In the heatmap, ALFC values shown are the mean ALFC value across development; invalid comparisons (NAs) are white/blank, highly variable genes are highlighted in red, and numbers indicate bootstrap support for the clustering analysis. **(C)** Convergence index values (see Methods) showing levels of convergence between *D*. *sec*. and *D*. *ere*. for antennal developmental transcription factors, averaged across development (T1–T3). Positive values indicate convergence while negative values indicate divergence. Error bars indicate S.E.M. **(D)** Plot of convergence index values versus absolute log fold change (ALFC) values for antennal developmental transcription factors. ALFC values shown are the mean ALFC value across all interspecies pairwise comparisons. The solid line shows a fitted line, with r as Pearson’s correlation coefficient.

We next extended our analysis to the developing olfactory system. The biology of the developing olfactory system is complex and the molecular determinants are not completely defined. Nevertheless, recent years have revealed the general logic and some of the molecular and developmental events generating the diversity of ORN fates and OR gene regulation in *D. melanogaster* ^15–17,20^. Thus, limiting our developmental analysis to TFs that have previously been identified as regulating ORN development^15–17,20,26,27^, we looked for differences in flexibility among TFs by comparing transcriptional variation, again using the pairwise interspecies ALFC values, for each TF across the three developmental time points. Similar to our adult analysis, we plotted the results both as a boxplot combining all pairwise comparisons and as a heatmap showing each interspecies comparison separately. In both the boxplot and the heatmap, we found markedly non-uniform patterns of interspecies variation in the transcript levels of TFs across all three developmental stages (Fig. 5A,B, see also Fig. S4 for expression levels across development for select genes). In all three TF categories, we detected high levels of transcriptional variation in only a few select transcription factors, while other transcription factor transcript levels were conserved. Additionally, similar to our adult analysis, clustering analysis of our heatmap revealed a subset of TFs with higher transcriptional variability than others, and both early-acting and late-acting factors were included in this subset (Fig. 5B). Thus, the results from these developmental analyses did not conflict with our results from the adult analysis, which suggested that developmental pathways for specific ORN lineages are more transcriptionally variable, and thus possibly more amenable to evolutionary change than others.

Next, to test the hypothesis that components with greater variability are more likely to be utilized during adaptive events in the context of development, we ranked the TF genes according to their convergence index as before (Fig. 5C). Again, high, positive values for this convergence index represent genes that are highly converged in *D*. *erecta* and *D*. *sechellia*, while low, negative values represent genes that are highly divergent in these two species relative to other species with equivalent divergent time estimates. These showed that, in contrast to OR genes, the vast majority of the TF genes included in the analysis had a positive convergent index value (Fig. 5C), highlighting the curious discrepancy between the extents of parallelisms observed during development versus in the adult olfactory system. Additionally, if components with greater variability are more likely to be utilized during adaptive events, we expected to find that TFs with the highest variation would also have high convergent index values. Indeed, among the TF genes, the TFs with highest convergence indices include many that we previously found to have relatively high transcriptional variation across species (Fig. 5B,C). Interestingly, many of these TFs were previously shown to specify the basiconic (particularly ab3) sensilla ORN fates^15,20^, which is the same sensilla class that we found to have the highest transcriptional variability in our adult analysis. Additionally, plotting gene variability versus convergence indices showed a statistically significant positive relationship between gene variability and convergence for TF genes, suggesting that highly variable TF genes are likely to be convergent between the two specialist species (Fig. 5D). This is in contrast to the null relationship between variability and convergence that we observed in OR genes, and supports the hypothesis that more variable developmental programs have a higher probability of being utilized during adaptive events. Our results also indicate that different sets of evolutionary constraints act on the developing and adult olfactory system.

### Relationship between variability in the developing and adult olfactory system

Given the combinatorial nature of the TF codes governing the specification of 50 ORN fates (i.e. a single ORN fate is regulated by multiple TFs, and a single TF controls many ORN fates), it is plausible to imagine transcriptional variation in one or combinations of TFs can lead to changes in many ORN fates. In this scenario, variation in the expression of specific OR genes or in the fate of some ORNs is a result of transcriptional changes in single or combinations of TFs in the developmental code for those fates.

**Figure 6:**
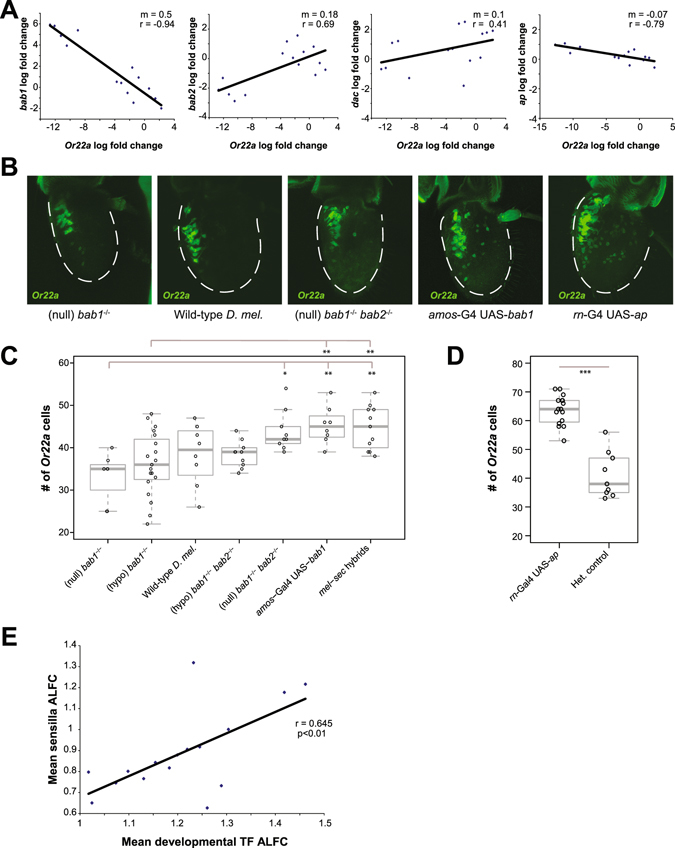
Relationship between highly variable TFs and highly variable ORs. **(A)** Plots of interspecies pairwise log fold change for Or22a against interspecies pairwise log fold change for pre-patterning TFs known to be involved in ab3 sensilla development. Solid lines indicate a linear regression, with m indicating the slope of the line and r indicating Pearson’s correlation coefficient. **(B)** Reporter analysis for *Or22a* expression in different mutant and overexpression backgrounds, arranged according to level of *Or22a* expression, using an *Or22a*-mCD8GFP promoter-fusion marker. **(C)** Quantification of changes in the number of *Or22a*-GFP cells when *bab1* and *bab2* expression is manipulated in different ways, with *sechellia-melanogaster* hybrids included for comparison. Statistical comparisons show results for ANOVA + Tukey’s HSD (*p < 0.05, **p < 0.01). **(D)** Increase in the number of *Or22a* GFP-positive cells when *ap* is overexpressed with a *rn*-Gal4 driver, compared to a heterozygous control lacking the Gal4 driver (p < 0.01, Student’s t-test, n = 9). **(E**) Variability of OR genes grouped by sensilla subtypes plotted against the variability of the transcription factors known to be involved in its development (see Table S1) shows positive correlation (Pearson’s coefficient = 0.645, *p* < 0.01, n = 16). Each point represents the mean ALFC for all OR genes from a sensilla subtype plotted against the mean ALFC during T1-T3 for all transcription factors known to be involved in the development of that sensilla subtype. The ab3 and ab4 sensilla were removed as mathematical outliers (see Methods).

We first asked whether there was a direct causal relationship between individual highly variable TFs and highly variable ORs. To do so, we decided to focus our investigation on *Or22a*, a highly variable OR gene whose expression levels and ORN appear to change in parallel in the two specialists, and the TFs known to be involved in its specification. Previous studies have shown that the transcription factors *dac*, *ap*, and *bab1*/2 are expressed in and/or are involved in the specification of the ab3 sensilla lineage that houses *Or22a* ^15,28^. By plotting the changes in these genes across species against changes in *Or22a* expression, we found *bab1* and *bab2* to be the top two predictors of *Or22a* variation, with inverse effects (Fig. 6A, also see Fig. S5 for a combined figure with other TFs).

To examine the relationship between *bab1/2* and *Or22a* in more detail, we analyzed single and double *bab* mutants in *D*. *melanogaster* using *bab1* and *bab2* mutant alleles with different strengths for *Or22a* ORN numbers. Given that both *bab1* and *bab2* show a decrease in expression in *D*. *sechellia* and *D*. *erecta* compared to *D*. *melanogaster*, these TFs might contribute to the changes in the representation of *Or22a* ORNs in the adult olfactory system in the specialists. We found that disrupting *bab1* and *bab2* levels in different combinations had variable effects on *Or22a* ORN numbers (Fig. 6B,C). Compared to the weak mutant alleles of *bab1*, which had very little to no effect on *Or22a* ORN numbers relative to wild type, stronger *bab1* mutant combinations trended towards lower *Or22a* ORN numbers. The increase in *Or22a* ORN numbers seen in *bab1-bab2* double mutants was similar to the number of *Or22a* ORNs seen in overexpression experiments with *amos*-Gal4 driving UAS-*bab1*, phenocopying the number seen in *D*. *sechellia/D*. *melanogaster* hybrids (Fig. 6C). In contrast, double mutants for *bab1* and *bab2* showed a statistically significant increase in *Or22a* ORN numbers when compared to *bab1* single mutants (Fig. 6C) (see also Methods for comparisons to *in situ* hybridization experiments), suggestive of an antagonistic regulatory roles for *bab1* and *bab2* in regulating *Or22a* ORN numbers. The inverse relationship observed in the regression analysis between the slopes for *bab1* and *bab2* effects on Or22a levels (Fig. 6A) also supports this antagonistic genetic relationship.

We also examined two other pre-patterning TFs thought to play a role in *Or22a* ORN development, *dac* and *ap*. Previous studies have shown that *dac* mutants have fewer *Or22a* ORNs and *dac* has been implicated in the regulation of basiconic sensilla identities^28^. *Dac* is one of the highly variable TFs and shows an increase in both transcript and protein levels in *D*. *sechellia* and *D*. *erecta* antennal discs (Fig. 3C, S4). To test the effect of high Dac levels on *Or22a*, we attempted to overexpress *dac* in *D*. *melanogaster* using *amos* ^*GAL4*^, *rn* ^*GAL4*^, and *dll* ^*GAL4*^; however, these all resulted in lethality at the pupal stage. Finally, we analyzed the transcription factor *ap*, which has been implicated as potentially being involved in the development of the ab3 sensilla^15^. *Ap* levels are only slightly variable across six species, suggesting that *ap* is a relatively conserved transcription factor. Interestingly, *Rn* ^*GAL4*^–mediated overexpression of *ap* resulted in a dramatic increase in the total number of *Or22a* ORNs (Fig. 6D).

Another hint for a link between variability in the TFs and OR variability was found when we looked for a broader correlation between adult OR transcriptional variability and TF variability by plotting the mean adult ALFC values for all of the ORs in each sensilla subtype against the mean developmental (T1–T3) ALFC values of all the known TFs required for their development. Interestingly, this comparison revealed a statistically significant positive relationship when ab3 and ab4 are excluded as outliers (Fig. 6E), a result that is consistent with the proximate cause of variation in some ORN lineages being transcriptional variation in the combinatorial TF code that specifies it. We, however, cannot fully explain all the variation in ORs by known TFs suggesting that there are unknown factors, such as undiscovered TFs affecting antennal development, that are contributing to variation in ORs. Overall, our results suggest that, rather than being caused by variation in any single TF gene, the variability of OR expression in the adult olfactory system is likely regulated by transcriptional changes in different combinations of multiple transcription factors during development.

## Discussion

The two host plant specialists, *D*. *sechellia* and *D*. *erecta*, have previously been shown to have parallel increases in the olfactory neuroanatomy responsible for detecting odors coming from their host plants^8,9^. Our results suggest that there is striking convergence in the transcriptional programs operating in the developing olfactory system of the two specialists compared to the other four generalist species. We also found non-random patterns of transcriptional variability for certain genes in the adult and developing olfactory system across all six species; a subset of antennal basiconic olfactory receptors, along with a subset of developmental transcription factors, show higher transcriptional variation across all six species compared to other genes in the same category. Greater convergence in transcriptional variability for TFs involved in olfactory system development in the two specialist species was also paralleled by greater interspecies transcriptional variability in those same TFs. Our results posit the hypothesis that, under the influence of ecological constraints, a limited number of variable olfactory receptors and the developmental regulators of the neurons that express them are more likely to both harbor variation and to change across evolutionary timescales, and thus contribute to adaptive processes.

However, our genetic data also suggest that single manipulations to TFs that show high evolutionary variation have only small individual effects on *Or22a* ORN representation. Thus, we were unable to find a conclusive link between variability in the TFs and OR variability through our genetics studies. This does not necessarily mean that such a link does not exist, as we only examined a single OR. Future work on identifying other TFs that contribute to individual ORN fates and the exact combinatorial TF code operating in each ORN fate will be necessary to rule out this hypothesis. This may also imply that the different configuration of ORN representation observed in *D. sechellia* and *D*. *erecta* are more likely the combinatorial result of simultaneous changes in many TFs, with large changes in highly variable TFs and small changes in more stable TFs.

The idea that some developmental genes may drive the evolution of ‘parallel’ physical traits in different natural populations is not new; previous research has highlighted individual developmental genes (such as *bab2* and *svb*) that drive specific convergent physical traits in different *Drosophila* species^29–33^. However, such evolutionary convergence has so far only been shown in the transcriptional regulation of a single gene driving a diversity of phenotypes for a given trait. Our results suggest that, in sensory systems with high neuronal diversity such as the *Drosophila* olfactory system, this pattern of convergence may extend to more complex developmental programs as well, such as the combinatorial transcription factor networks that define the ORN fates in the *Drosophila* olfactory system.

Our results also suggest that in the *Drosophila* olfactory system, different sets of evolutionary constraints may apply during development and in adults, leading to differences in the extent of parallelisms observed at the two phases. We hypothesize that this may be because the combinatorial code allows for robustness during development — that is, the combinatorial code can buffer many changes in the local expression of specific transcription factors without greatly affecting the final adult phenotype^34^. In contrast, the expression of adult ORs directly contributes to adult olfactory behavior and may thus be under more stringent selective pressure. This would account for our observed increase in levels of convergence across the developmental stages compared to the adult stage. Alternatively, due to the combinatorial nature of ORN specification, certain ORN lineages might be more susceptible to changes in the code. For example, one would speculate that ORN lineages generated by combinations containing the highest number of variable TFs would show the highest variation, whereas combinations with TFs that show little change, or with fewer variable TFs, would not affect adult ORNs. Which of these scenarios is true remains unknown and only a more detailed molecular understanding of ORN development will answer this question.

Our data also suggest that flexibility in the *Drosophila* olfactory system may be associated with specific ORN developmental trajectories, particularly those from basiconic sensilla. That is, a specific subset of the olfactory system tolerates more variation and as a result is more free to diverge over time. Indeed, these results are consistent with our earlier hypothesis that large and thin basiconic ORN fates are likely to be evolutionarily variable due to the irregularity of precursor fate decisions for diverse subtypes in these sensilla classes^23^. This observation, in turn, suggests that there are specific pathways in both the developing and adult olfactory system, which are less “central” (i.e. not as important) to the development or function of the olfactory system and thus more amenable to change, which is broadly consistent with other studies of the *Drosophila* olfactory system^35^. This may also be related to the pleiotropic principal, which posits that genes with greater pleiotropy (multiple functions) tend to be more stable or conserved^36^.

At this point our data is suggestive, not conclusive; however, we believe that our work suggests several explicit tests of this idea that can be addressed with future work. For example, we predict that *bab1* and *bab2* may be important players in the evolution of ORN differences among species. As ORN developmental trajectories are defined, many TFs act as binary switches shifting the fate of a cell from one ORN to another. A few TFs instead act as gradients. *Bab1* and *bab2* are expressed in a gradient within the antennal disc. Both of these genes show high transcriptional variation across six species, and in case of *bab1*, shows convergence between specialists. *Bab1* and *bab2* also belong to the gene regulatory network that patterns the larval antennal disc into concentric discs with diverse differentiation potentials^15^. The expression of both genes starts at the latest stages of antennal disc patterning, and are among the latest known molecular components to be added to the gene regulatory network^26^, perhaps acting as a node that has not completely consolidated and remained variable during evolution. Gradient genes like *bab1* and *bab2* may be an alternative manifestation of the pleiotropic principle: genes with graded expression within a tissue might be phenotypically more robust to changes in transcription, as nucleotide changes in regulatory elements only change levels within the gradient or patterns, not wholesale switches in fate. This could lead to a subset of the system that is more robust to harboring genetic variation than other developmental genes that have all-or-none functions and thus more likely to contribute to evolution. Further work will be required to see if any of these factors, individually or in combination, contribute to the evolution of developmental traits in the peripheral olfactory system.

There are, of course, alternative explanations for our results that we cannot yet rule out. For example, it is possible that the similarities we find between *D*. *erecta* and *D*. *sechellia* are due to biogeographical similarities, rather than host-plant specialization. However, because the geographical distributions for many of these species overlap extensively, we believe that biogeographical similarities between *D*. *erecta* and *D*. *sechellia* are less likely to drive the patterns we observe than host-plant specialization. In addition, previous studies have suggested that the ancestral state for the entire *melanogaster* subgroup is equatorial (further, *D*. *simulans* is also found on the Seychelles, where *D*. *sechellia* originated)^37^. Thus, biogeography should be driving similarity in the entire clade, not just in the two host-specialists.

Another possible explanation is that we just happened to samples strains that showed this pattern and that a wider sampling of each taxa would show some individuals are “parallel” and others are not. However, previous studies that have examined the adult olfactory transcriptome of multiple species have found variation between different strains of the same species to be small enough as to not significantly affect comparisons between species^38^, although ideally future studies should include comparisons from additional strains. In addition, a brief comparison of *Or22a* expression in different *D*. *mel*. backgrounds did not show any significant differences, and also suggested that differences between individuals of the same species are about 20 percent, compared to differences between species which can be an order of magnitude or more (Fig. S6).

The idea that developmental systems cannot be generically flexible — that is, all components are equally likely to evolve — but that there are flexible components within these systems is also not new; as far back as 1940, Goldschmidt speculated that “paths are available for changes in the genotype…without upsetting normal developmental processes”^39^. Our data suggests both that these paths exist and that they are not idiosyncratic. Instead, these evolutionary “lines of least resistance”^40^ tend to be conserved over time, which suggests that these flexible components may be hotspots of phenotypic evolution. The idea that specific parts of the olfactory system are more amenable to evolutionary change is also consistent with a recent study on the evolution of the *Drosophila* olfactory system that highlighted ester-sensing receptors as being particularly evolutionarily malleable^41^, as well as with other studies that have highlighted basiconic sensilla, particularly the ab3 sensilla, as having diverged either in sequence, expression, or function under various different circumstances across the *Drosophila* genus^7–9,25,38,42–45^. Thus, it is likely that large basiconic sensilla, which house *Or22a*, are a developmental hotspot that has been repeatedly utilized for novel functions during evolution.

Finally, our results also indicate that variation in highly variable ORs is associated with changes in the underlying combinatorial developmental code, and transcriptional variability in adult sensilla subtypes is correlated with transcriptional variability in their developmental regulators. The basiconic sensilla appear more evolutionarily labile, which suggests that we may be able to predict, in certain cases, some of the molecular differences that cause species-specific behavioral adaptations if the variability of the developmental regulators underlying the system is known. Thus, we propose that, to a limited extent, transcriptionally variable developmental programs may be able to act as predictable hotspots of evolution in a sensory system with high neuronal diversity, providing a limited set of possible configurations that drive the evolution of ‘parallel’ physical traits in different natural populations undergoing similar selective pressures^46^. Defining the distinctive characteristics of gene expression within developmental programs in other systems will ultimately determine if such patterns can be used as predictors of how organisms evolve.

## Materials and Methods

### Fly strains, rearing, and collections

The six *Drosophila* species genotypes we used were *D*. *ananassae* (14024-0371.00, San Luis Potosi, Mexico, *Drosophila* Species Stock Center (DSSC), University of California, San Diego), *D*. *erecta* (14021-0224.00, DSSC), *D*. *sechellia* (14021–0248.01, DSSC), *D*. *simulans* (14021-0251.165, Florida City, Florida, DSSC), *D*. *virilis* (15010-1051.00, Pasadena, California, DSSC) and the*w* ^1118^ strain of *D*. *melanogaster*. All flies were reared on cornmeal medium using a 16:8 light:dark cycle at 20 °C.

For RNA extraction, ~70 wandering third instar larvae antennal discs, ~70 8hr APF pupal antennal discs, ~70 40hr APF pupal antennae, and ~300 adult antennae (150 males and 150 females) from each species were collected. Pupal antennal discs/antennae were dissected from pupae that had been collected as white pupae and placed in a 25 °C incubator for 8 or 40 hours (*D*. *melanogaster*, *D*. *sechellia*, and *D*. *erecta*), or for 10 or 48 hours (*D*. *virilis*). Timing for the pupal dissections of *D*. *virilis* was based both on the observation that *D*. *virilis* pupal development at 25 °C was roughly 1.3 times longer than the other species, and on comparative observations of antennae/antennal disc morphology during dissections, with priority given to morphological considerations to be as identical as possible. Adult antennae were collected from adult flies that were 7–10 days post-eclosion.

### Sample preparation and RNAseq

We extracted RNA using the RNeasy kit (Qiagen) according to the manufacturer’s instructions, including an on-column DNase digestion (Qiagen). RNA quality and concentration were measured using 700 ng RNA diluted to 60ul total volume. RNA sequencing libraries were prepared with TruSeq Stranded mRNA Sample Prep Kit (Illumina) according to manufacturer’s instructions. For the RNA fragmentation step, 94 °C, 2 min was used with the intention to obtain a median size ~185 bp. PCR amplification was done with 15 cycles. Libraries (barcoded) were multiplexed in sets of 24. Each set was accessed for quality before separation into two identical pooled libraries, which were then subjected to cluster generation followed by 50 bp paired-end sequencing with an Illumina HiSeq 2500 sequencer by the UNC High-Throughput Sequencing Facility (HTSF).

### Alignment, mapping and generating gene counts

Basecalls were performed using CASAVA version 1.8. Sequenced reads were trimmed for the adaptor sequence, and then mapped to each species’ transcriptome that was downloaded from FlyBase (FB2014_6 release) using bwa-0.7.8. Depending on the species, 50–90% of the total reads were successfully mapped with the reference transcriptome. Count tables for each sample were generated using a customized python script, and each transcript ID was given a gene identifier based on ortholog calls from FlyBase. These orthology calls were then confirmed by reannotating the gene models from the raw read and performing comparative BLASTs. While minor discrepancies were detected in the gene models, most annotations were supported. The count tables were then consolidated into matrices containing transcript ID and read counts from all genotypes for each stage using a Ruby script. For genes with multiple orthologs, only the highest expressed transcript was included for comparison. Genes without orthologs in one or more species were listed as NAs for those species. Matrices of gene counts were normalized for each species’ developmental time points in R (3.1.2) using the DESeq. 2 suite (release 3.4). After estimating the dispersion parameter, we fit the data and tested for significance using a negative binomial test with the following model: *expression = ~spp*. FDR was controlled for using Benjamini-Hochberg method. All scripts used in the pipeline are available from the authors. Raw sequence data are available for download from the NCBI Gene Expression Omnibus (GEO) database using the accession numbers GSE85239 and GSE75986, and the normalized count tables are also available in the GSE85239 series.

### Data analysis

The variability of a gene was determined by calculating the mean absolute log fold change for all pairwise species comparisons. Absolute log fold change (ALFC) values were calculated by comparing DESeq-normalized transcript counts for each species to every other species in a pairwise fashion, and taking the absolute of the log fold change in transcript counts to provide a non-directional estimate of the difference in gene expression between each pairwise species comparison for every gene. For whole-antennal transcriptome analyses, only genes with transcript counts above 50 in all species were included for comparisons. For analyses using specific lists of olfactory receptor or transcription factor genes, matrices were curated by hand to remove genes with low or no expression across all species (<30 transcript count).

### Heatmaps and cluster analysis

Heatmaps were created in R using the heatmap.2 function from the gplot package (v3.0.1). Invalid comparisons (NAs) where one of the species does not have a comparable ortholog, or the ortholog is pseudogenenized, are indicated with white/blank regions. Cluster analysis was done with the default clustering options from the heatmap.2 function that uses hierarchical clustering with the distance and cluster methods set as “euclidean” and “complete”, respectively. Bootstrap analysis of clusters was done using the pvclust package in R (2.0-0) for 10,000 iterations.

### Correlation between transcription factor and sensilla subtype variability

The variability of each sensilla subtype was defined as the mean ALFC value across all pairwise comparisons for all ORs in that sensilla subtype, and plotted against the variability of the transcription factors known to be involved in its development, defined as the unweighted mean ALFC value for all TFs with known involvement across all pairwise comparisons and developmental time points. The fitted line for these values is shown, and the *r* value indicated is Pearson’s coefficient. Given the sensitivity of this type of analysis to outliers, the ab3 and ab4 sensilla were excluded from analysis as mathematical outliers (>1.5 interquartile ranges above the first quartile).

### Correlation between convergence indices and gene variability

The variability of each gene was defined as the mean ALFC value across all pairwise comparisons for all species, and plotted against the convergence index values as defined above. The fitted line for these values is shown, and the *r* value indicated is Pearson’s coefficient. Given the sensitivity of this type of analysis to outliers, the *Or33a* and *Or85b* genes were excluded from the analysis of adult OR genes as mathematical outliers (>1.5 interquartile ranges above the first quartile).

### Phylonormalization

Some analyses were phylo-normalized, where the mean ALFC values were adjusted to be equivalent between closely- and distantly-related pairwise species comparisons according to a linear model of ALFC values versus phylogenetic distances taken from literature^2^.

### Principal components analysis

PCA was done using the AMOR package (AMOR_0.0-15) in R with default options.

### Convergence index

The ranking of genes according to the strength of convergent expression between *D*. *sechellia* and *D*. *erecta* was done by finding the difference between the Sec-Ere ALFC value and the mean of the Sim-Ere and Mel-Ere ALFC values (the larger the difference the higher the rank), to in essence find similarly expressed genes normalized by the other species with equal phylogenetic distance. Thus, a highly ranked gene would be one that was very similar between *D*. *ere*. and *D*. *sec*. but very different from *D*. *mel*. and *D*. *sim*.

### Genetics

To determine the effects of manipulating *bab* expression on *Or22a* expression, we used *D*. *mel*. flies with an *Or22a*-mCD8GFP promoter-fusion construct on the X chromosome on a *w1118* background^11^. We crossed these flies to flies with different combinations of *bab* mutant alleles. In order of increasing *bab* expression (decreasing strength of bab mutant phenotype), these alleles were *bab* ^*DFPA*^, *bab* ^*A07*^, *bab* ^*PR72*^, *bab1* ^*Gal4*^, and *bab1* ^*A128*^, respectively^15^. The former three alleles affect both *bab1* and *bab2* expression while the latter two are *bab1*-specific. Utilizing different allelic combinations allowed us to determine the response of *Or22a* to different levels of *bab1* and *bab2* expression. We also used *amos*-Gal4 driving UAS-*bab1* to test the results of *bab1* overexpression on *Or22a* expression. As for our *ap* manipulations, we used UAS-*ap* driven by *rn-*Gal4 on the same *Or22a*-mCD8GFP promoter-fusion background^15^. *Dan* mutant heterozygous flies were characterized in a previous study^27^.

### Reporter analysis in hybrid crosses

Hybrid *melanogaster-sechellia* or *melanogaster-simulans* flies were generated by crossing female *D. melanogaster* flies homozygous for the OR promoter-fusion construct with wild-type male *D*. *sechellia* or *D*. *simulans* and imaging the F1 generation. The direction of these interspecies cross results in only females being produced, thus it is important to note that our hybrid analyses examine only females flies, contrary to most of our other experiments that included both genders. The OR promoter-fusion constructs used were *Or42b*-mCD8GFP and *Or92a*-mCD8GFP that have been characterized in previous studies^15,23^.

### Real-time RT-PCR

Antennae or antennal discs from approximately 100 flies or 50 larvae, respectively, were dissected and analyzed for each species. RNA was extracted with an RNeasy kit (Qiagen), treated with on-column DNase digestion (Qiagen), and then reverse transcribed into cDNA using the SuperScript First-Strand Synthesis System for RT-PCR (Invitrogen). qPCR was performed with the FastStart Universal SYBR Green Master Mix (Roche) using standard protocol. Expression for each gene was analyzed in triplicate. RNA concentration was standardized to 15 ng/µl before reverse transcription, and cDNA was diluted 1:32 before use. Ct values were normalized to each species’ Actin 5 C (Act5c) expression. Primers used in the reactions are listed in Table S2.

### Immunohistochemistry

Samples were fixed with 4% paraformaldehyde, washed with phosphate buffer with 0.2% Triton X-100, and stained as previously described^23^. Primary and secondary antibodies were used in the following dilutions: mouse anti-Dac 1:20 (Developmental Studies Hybridoma Bank), goat anti-mouse-Cy3 1:100, rat anti-Bab2 1:1500 (Frank Laski)^47^, goat anti-rat-Cy3 1:200. Confocal images were taken by an Olympus Fluoview FV1000.

### *In situ* hybridization

Digoxigenin RNA probes were made using a Roche DIG RNA labeling kit (Indianapolis, IN, USA). *Drosophila* heads were dissected into cold fixative (4% paraformaldehyde, 0.05% Tween 20 in 1X PBS) and fixed for one hour. Heads were then washed 3 × 10 min in PBST (1X PBS, 0.1% Tween 20). The third antennal segment was dissected and fixed for an additional 30 min. Tissue samples were washed 5 × 5 min in PTX (1X PBS, 1% Triton X) and incubated in hybridization (Hyb) buffer (50% formamide, 5X SSC, 0.05 mg ml^−1^ heparin, 0.1% Tween 20) for two hours at 55 °C, before hybridization overnight at 55 °C with DIG-labeled RNA probe. Tissue was then washed 5 × 20 min in Hyb buffer at 55 °C, with the last wash proceeding overnight. Samples were then washed for 20 min in Hyb buffer at 55 °C, followed by 5 × 5 min washes in PBST at room temperature prior to incubation for 3 h in 1:500 anti-DIG-AP in PBST and 1X BSA. Next, samples were washed 5 × 5 min in PBST and incubated in FastRed solution (Roche, Indianapolis, IN, USA) for 30 min. Samples were washed for a final 5 × 5 min in PBST and stored overnight in mounting solution before imaging.

### Data availability

Raw sequence data are available for download from the NCBI Gene Expression Omnibus (GEO) database using the accession numbers GSE85239 and GSE75986, and the normalized count tables are also available in the GSE85239 series. All other data, including any scripts used, are available from the authors.

## Electronic supplementary material

Supplementary figures and tables
