# Supplementary material for: Patterns of transcriptional parallelism and variation in the developing olfactory system of Drosophila species
Source: Sci Rep. 2017 Aug 18;7:8804. doi: 10.1038/s41598-017-08563-0 (PMC5562767; doi:10.1038/s41598-017-08563-0)
Supplement: Supplementary file 1 — Supplementary figures and tables [file 41598_2017_8563_MOESM1_ESM.pdf]

Supplemental Information

for

Patterns of transcriptional parallelism and variation in the developing olfactory system of *Drosophila* species

Jia Wern Pan, Qingyun Li, Scott Barish, Sumie Okuwa, Songhui Zhao, Charles Soeder, Matthew Kanke, Corbin D. Jones, Pelin Cayirlioglu Volkan

Supplemental Figures

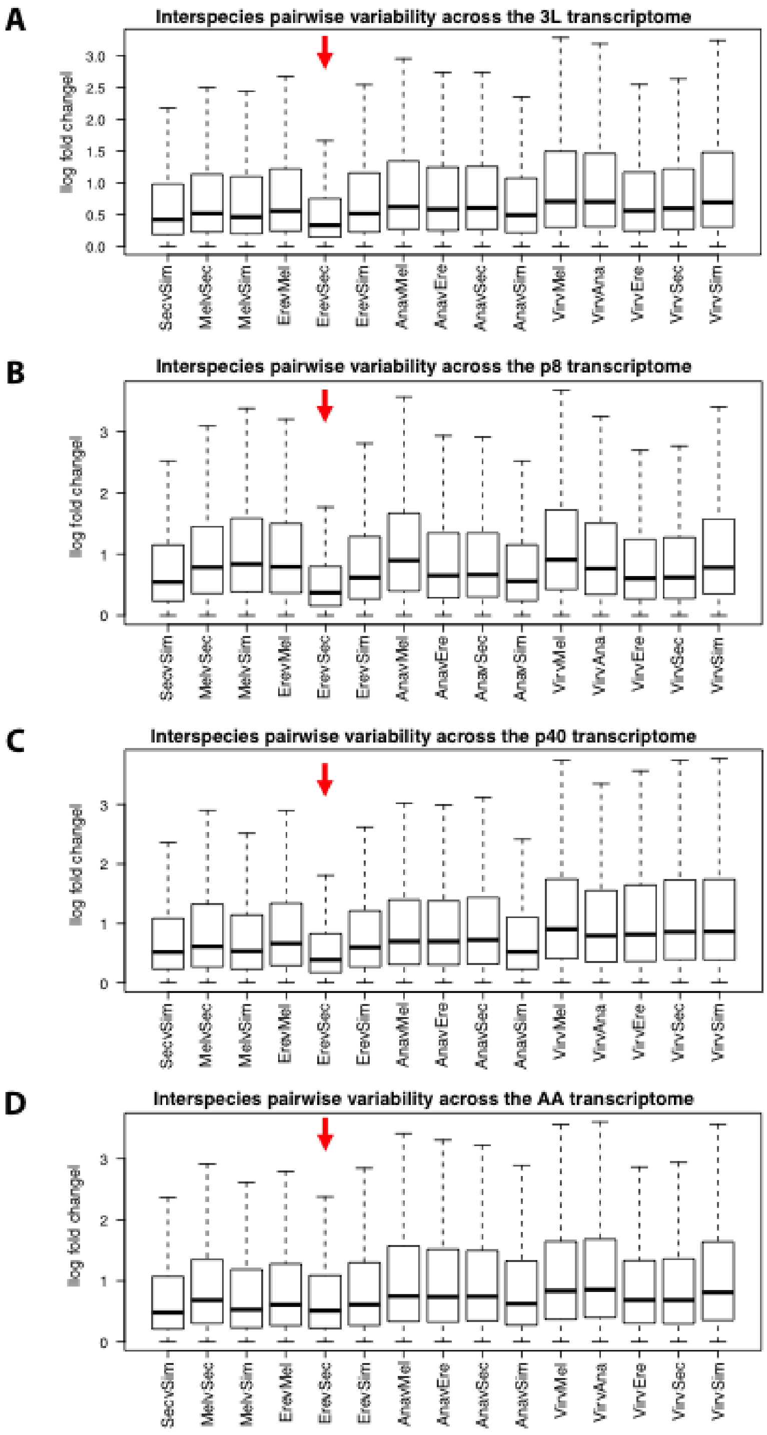

Fig. S1. Antennal transcriptome similarities between *D. erecta* and *D. sechellia* are strongest during development. Pairwise interspecies comparisons of absolute log fold change in expression across the whole antennal transcriptome during the **(A)** 3rd instar larvae, **(B)** 8hr APF, **(C)** 40hr APF, and **(D)** adult stages indicate that the similarities are most apparent during the developmental stages (A, B, C) but less apparent in the adult antennae (D).

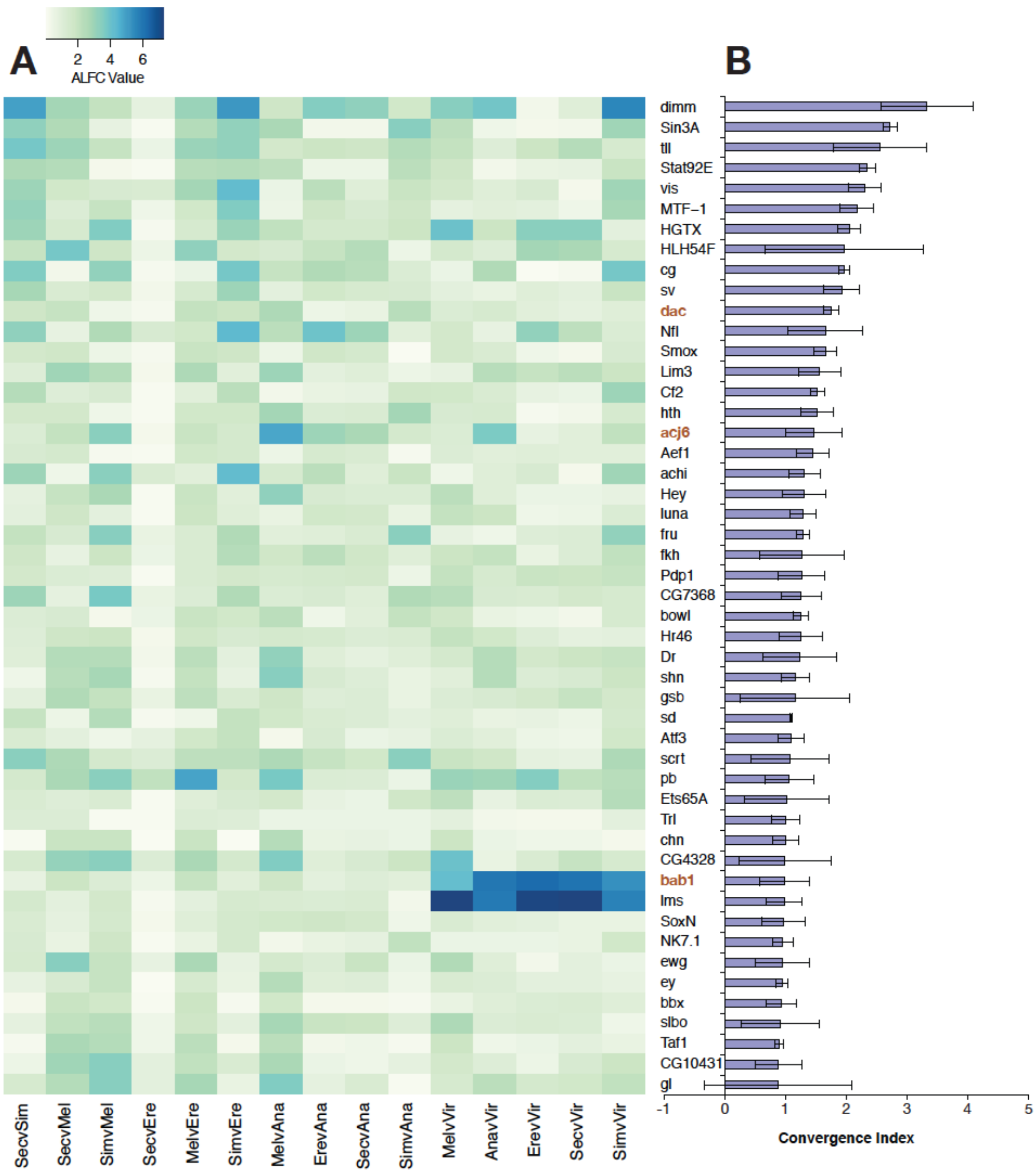

Fig. S2. Transcriptional variability and convergence index of 50 transcription factors. (A) Heatmap of absolute log fold change (ALFC) values for 50 transcription factor genes showing the most convergent transcription between *D. erecta* and *D. sechellia* out of 250 known transcription factors. ALFC values shown are the mean value across development. Highlighted in red are transcription factors previously shown to be involved in antennal development. Note also the low ALFC values across the SecvEre column. **(B)** Convergence indices (see Methods) for the 50 genes that show the highest values out of 250 known TFs. Error bars indicate S.E.M.

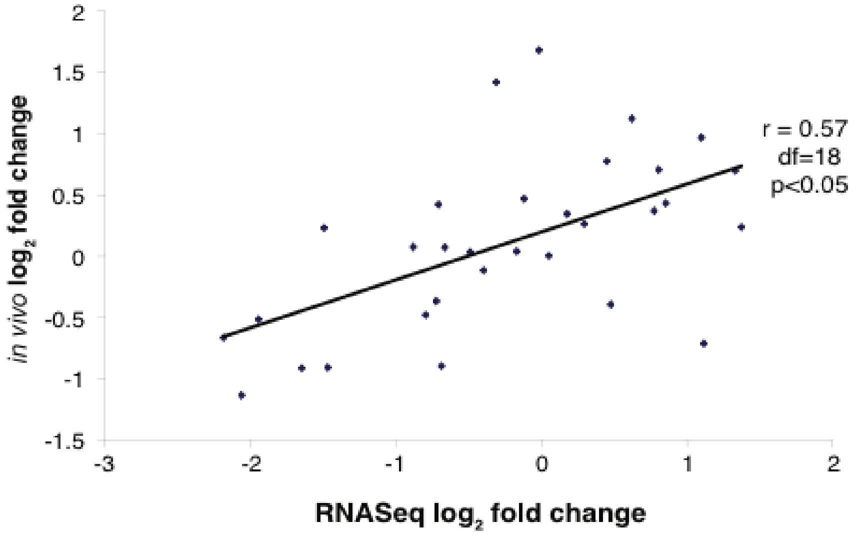

Fig. S3. Correlation between RNASeq and *in vivo* results. Interspecies pairwise log fold change in gene expression as for *Or22a*, *Or47a*, *Or47b*, *Or92a*, *Or42a*, *bab1*, *bab2*, and *dac* as measured by RNAseq plotted against the corresponding interspecies pairwise log fold change in quantifications of the *in vivo* experiments shown in Fig. 3. The solid line indicates a fitted line, with r as Pearson's correlation coefficient ($r=0.566$, $df=18$, $p<0.05$).

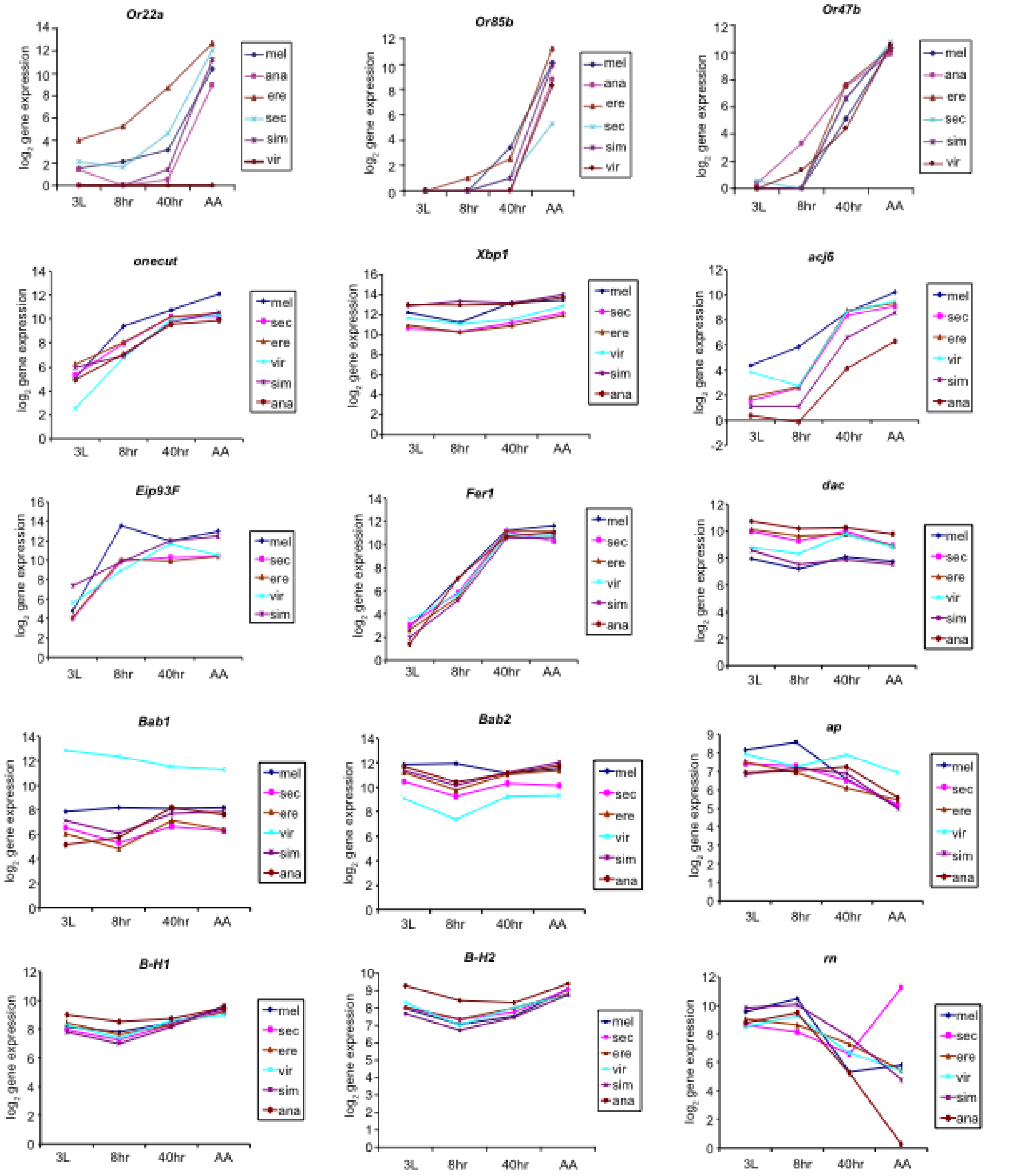

Fig. S4. Developmental expression profiles of select genes. Log_2- transformed DESeq-normalized transcript counts for select genes for each species across the four developmental time points. Error bars are omitted for readability.

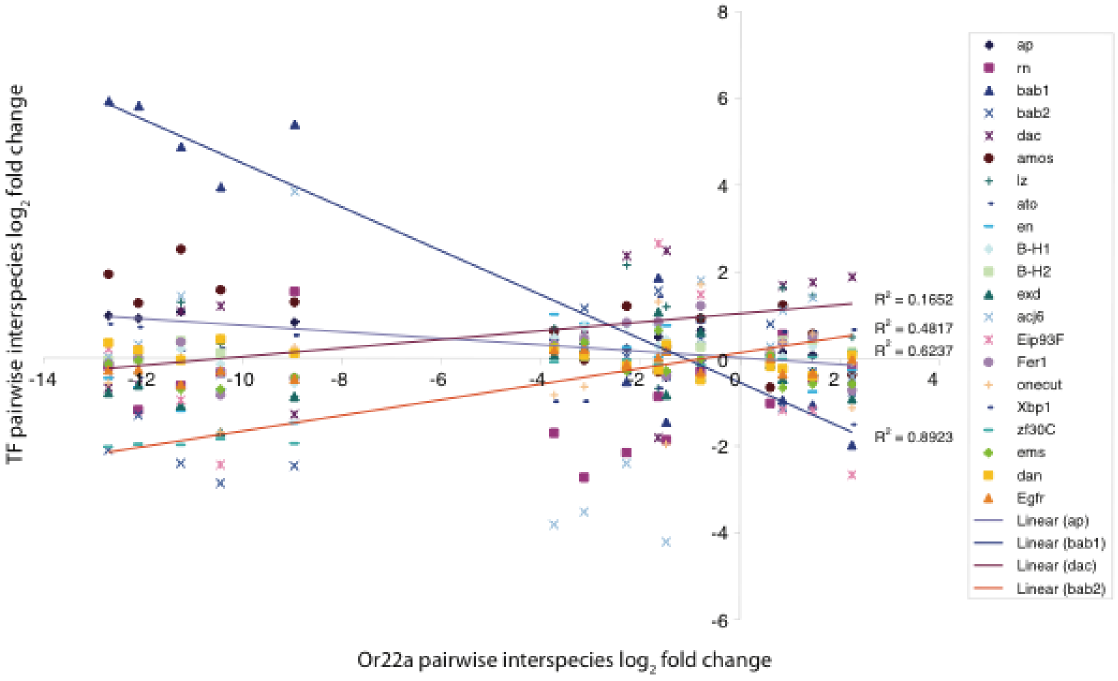

Fig. S5. Bab1 is a strong negative predictor of changes in *Or22a* expression. Plot of interspecies pairwise log fold change in expression of *Or22a* on the x-axis compared to log fold change in expression for select developmental transcription factors on the y-axis indicate a particularly strong negative relationship between *bab1* and *Or22a* expression. *Bab2*, on the other hand, has a positive relationship with *Or22a*. Other TFs with weaker relationships include *ap* and *dac*, which are also known to influence the development of large basiconic sensilla.

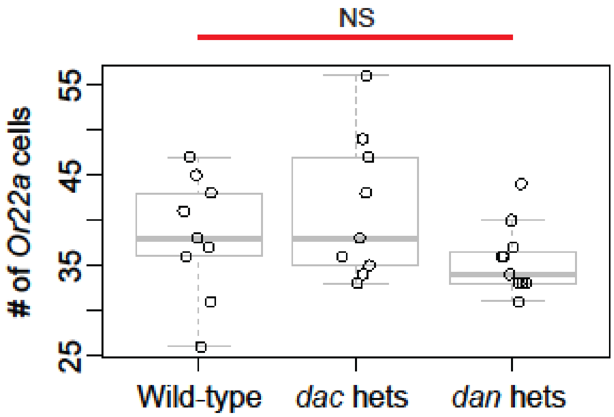

Fig. S6. *Or22a* expression in different *D. melanogaster* backgrounds. Boxplots showing the number of cells expressing an Or22a-mCD8GFP promoter-fusion marker in *D. melanogaster* wild-type background, *D. melanogaster dac* heterozygous mutant background, and *D. melanogaster dan* heterozygous mutant background. ANOVA + Tukey's HSD tests did not find any significant differences.

Supplemental Tables

Table S1. List of sensilla subtypes and corresponding transcription factors with known* developmental involvement

Sensilla subtype	Transcription factors with known involvement
ab1	amos,lz, dac, acj6, fer1, onecut, sim, xbp1, zf30c
ab2	amos, lz, bab, ap, acj6, fer1
ab3	amos, lz, bab, dac, acj6, fer1, onecut, xbp1, zf30c
ab4	amos, lz, bab, ap, acj6, fer1,zfc30
ab5	amos, lz, rn, bab, acj6,e93
ab6	amos, lz, bab, ap, sim
ab7	amos, lz, rn, bab, dac, acj6, xbp1
ab8	amos, lz, bab, ap, acj6, fer1, zf30c
ab9	amos, lz, dac, acj6, onecut
ab10	amos, lz, rn, dac, acj6, e93, fer1, onecut, xbp1, zf30c
at1	amos, lz, rn, bab, zf30c
at2	amos, lz, bab, ap, e93, fer1, xbp1
at3	amos, lz, rn, bab, acj6, e93, fer1, onecut
at4	amos, lz, bab, bar, ap, e93, fer1
ac1	ato, rn, bab, bar, ap, acj6, xbp1
ac2	ato, bab, bar, ap
ac3	ato, bab,ap, acj6, fer1, zf30c
ac4	ato, rn, bab, bar, ap

*References: Jabari et al. 2013, Li et al 2015, Gupta and Rodrigues 1997, Goulding et al. 2000

Table S2. List of primers used for qPCR validation

Primer Name	Sequence
ACT5C-qPCR-F	GGCGCAGAGCAAGCGTGGTA
ACT5C-qPCR-R	GGGTGCCACACGCAGCTCAT
Dmel_bab1_qPCR_F	AGGAATGTAAGAAAGCGCCG
Dmel_bab1_qPCR_R	CGAACAGTCCCTCTGTCTCT
Dmel_BarH1_qPCR_F	TTTGTAGCAATGGCGGTAAAGA
Dmel_BarH1_qPCR_R	CAGCTGGTGATCCGTGAAGG
Dmel_dac_qPCR_F	GTCTGCAATGTGGAACAGGT
Dmel_dac_qPCR_R	CTTGATCGATAGGCATCTGGC
Dmel_Or22a_Forward	GCGGGGGAGTTCCTTAGTTC

Dmel_Or22a_Reverse	CCTGGCTTCGATCGGAGATT
Dsim_Or47b_qPCR_F	CCTTTCACTGCGTCTATCCG
Dsim_Or47b_qPCR_R	GATATGCCCACCATGTCCAC
Dsim_orco_qPCR_F	CCACTGCATCACGAAGTTCA
Dsim_orco_qPCR_R	TTCCTCATCTTTGCCAGTGC
GAPDH2-qPCR-F	CGTTCATGCCACCACCGCTA
GAPDH2-qPCR-R	CCACGTCCATCACGCCACAA
Gr21a-qPCR-F	CCAACATGTACGGCATGTACT
Gr21a-qPCR-R	ACAGACCCACCTCCTTGTAG
Or22a-qPCR-F	CCGATCGTCGCTACAAATCC
Or22a-qPCR-R	ATGCCAGCTTCACCATAGCC
Or47b-qPCR-F	CAAATCTCAGCCTTCTGCGG
Or47b-qPCR-R	GATACTGGCACAGCAAACTCA
Or98a-qPCR-F	ATTCAAGCCGCAGTTACAAGT
Or98a-qPCR-R	TGCCAGCTTAGCCACCTTAAT
qPCR_Dmel_elav_F	CACACCGAGCGAAATACGG
qPCR_Dmel_elav_R	TTCTCCCACACTCTCTTCGG
qPCR_Dmel_Orco_F	GCCTAGATGATTGCTGCATTACT
qPCR_Dmel_Orco_R	CGAGGTTGTCATCCTTGCTATT
qPCR_Dsech_elav_F2	CGAGATTGAGTCGGTGAAGC
qPCR_Dsech_elav_R2	AACATTAACAGCCTGCTCGG
qPCR_Dsech_orco_F	GTCTACGCCTTAACAGTCGTC
qPCR_Dsech_orco_R	CGGATGAACTCTCTTCAATCAGG
Dana_Act5C_qPCR_F	CGCCATTCTCCGTCTTGACT
Dana_Act5C_qPCR_R	ATCACGGACGATTTCACGCT
Dana_bab1_qPCR_F	TCGGCTTGCTCTCCATACTT
Dana_bab1_qPCR_R	ACTCCACAATGGCCTTCAGA
Dana_BarH1_qPCR_F2	CAGCGACTATCACGAGGAGA
Dana_BarH1_qPCR_R2	TGATGCTGTTTCCGTCATCG
Dana_dac_qPCR_F	AAGCCACCAGAGAGTTCCAG
Dana_dac_qPCR_R	CCCATTATCCAGACGGTGTTTC
Dana_elav_qPCR_F	AGAGCTTTGATTGCCGAGTG
Dana_elav_qPCR_R	TGGCTCCGTTATTTGCCATC
Dana_Gapdh2_qPCR_F	TGTTGAGTCGACTGGTGTCT
Dana_Gapdh2_qPCR_R	CAGACGAACATGGGAGCATC
Dana_Gr21a_qPCR_F	CTCTGCAGATGACCATTCGG
Dana_Gr21a_qPCR_R	CCAGGCAGTACATGCCATAC
Dana_Or22.3_qPCR_F	TCGATGGTGACTGAGTACGTT
Dana_Or22.3_qPCR_R	CCTTGAACTTTGTGAAGCCCA
Dana_Or47b_qPCR_F	CGAGATGAGGGCTGTAAACG
Dana_Or47b_qPCR_R	TCAATTGGGCGTTGAACGAT

Dana_Or98a_qPCR_F	TACCCTGGCGCATTTACAAC
Dana_Or98a_qPCR_R	GGTGACAGCAAAGAGCATGA
Dana_orco.2_qPCR_F	GAACGGAGCTAATCCCAACG
Dana_orco.2_qPCR_R	AACATGTGCAGCAGTAGAGC
Dere_bab1_qPCR_F2	CACATCAAGGGCGTCATCAA
Dere_bab1_qPCR_R2	CTGGATAGCTGAGAGGGAGG
Dere_BarH1_qPCR_F	ACAAGTTGGACCTGAGCGAT
Dere_BarH1_qPCR_R	AAGGTGATCCGCCGTAAAGT
Dere_dac_qPCR_F	AGCCACCAGAAAGTTCCAGAC
Dere_dac_qPCR_R	GTTGTCCAGGCGATGCTTCTT
Dere_Or22a_Forward	TCGGAGTCAACATGTACGGC
Dere_Or22a_Reverse	TCGGTGAGCTCCGACAAGTA
qPCR_Dere_Act5C.2_F	CGAGTGGTGGAAGTTTGGAG
qPCR_Dere_Act5C.2_R	ATACGCTGGAACCACACAAC
qPCR_Dere_elav_F	GTTAAAGAGATCGGGAGAAGCAAA
qPCR_Dere_elav_R	TGACCTTTCTTTCACAGTGGC
qPCR_Dere_GapDH2_F	AGTTCGTGAAGCTGATCTCTTG
qPCR_Dere_GapDH2_R	GGTGGTTGCTTGGTGTTTCAC
qPCR_Dere_Gr21a_F	CAAGAGCCTTCTGGTGCTTT
qPCR_Dere_Gr21a_R	CAGCTGTAGATGAAGACGGC
qPCR_Dere_Or22a_F	TCCCTTCATCGACTCCGAAA
qPCR_Dere_Or22a_R	GCAGGTTGATGTGGCACTTA
qPCR_Dere_Or47b_F	GTGGGCATTTCCACCTTTCT
qPCR_Dere_Or47b_R	GAAGCGAACCACTCGACAAA
qPCR_Dere_Or98a.1_F	TTCTGACTTCACCAGAGGCA
qPCR_Dere_Or98a.1_R	TGATGATTCCGATGGGCAGA
qPCR_Dere_Orco_F	AAGACCTTCGTCCAGATCGT
qPCR_Dere_Orco_R	AGAACCGAGGCAAACAAGTC
Dvir_bab1_qPCR_F2	CGGATGTCACCCAAATGGAG
Dvir_bab1_qPCR_R2	AATTCTTGGGCGAATGCTGT
Dvir_BarH1_qPCR_F	GTTGCCACATCATTCGCATTC
Dvir_BarH1_qPCR_R	TGGCTTCAAATTGGACGAGG
Dvir_dac_qPCR_F	GCGGCAGTGATAAGTCTGAG
Dvir_dac_qPCR_R	GCTCGCGTTTAAAGTGGGTA
Dvir_Or98a_qPCR_F3	GTTGCGTCAAGGATGAGGAG
Dvir_Or98a_qPCR_R3	ACCACCGAGGTCAAATAGGT
qPCR_Dvir_Act5C.2_F	AAACGTGGTATCCTCACCCT
qPCR_Dvir_Act5C.2_R	TAGAACTGGATGCTCCTCGG
qPCR_Dvir_elav_F	CTAAGCGCAACAACAATGCCT
qPCR_Dvir_elav_R	AGACGCGATTTGAAGCCAGA
qPCR_Dvir_GapDH2_F	AAACTTACCGGCATGGCTTT

qPCR_Dvir_GapDH2_R	AGCCTCTTGTACTTTGGCCT
qPCR_Dvir_Gr21a_F	ATGCATTCTATCGTGCCACC
qPCR_Dvir_Gr21a_R	TGCTTGGATGTCCAGGTGTA
qPCR_Dvir_Gr63a_F	TATGCCTCGGTTAATGTGCG
qPCR_Dvir_Gr63a_R	CGCAATTGATGTTCGAGGGA
qPCR_Dvir_Or22a_F	TTTCACAACGGTGCTTGGAA
qPCR_Dvir_Or22a_R	ACACTTGTAGCGAGGTGAGA
qPCR_Dvir_Or47b_F	ACCACACCGAAATTCATCCG
qPCR_Dvir_Or47b_R	GCTGTGTGGAGAAAGGAGTG
qPCR_Dvir_Orco_F	CAAGTATTGGGTGGAGCGTC
qPCR_Dvir_Orco_R	GATAAGCCAGCAGAGTGAGC
